# Cutaneous Disseminated and Extracutaneous Sporotrichosis: Current Status of a Complex Disease

**DOI:** 10.3390/jof3010006

**Published:** 2017-02-10

**Authors:** Alexandro Bonifaz, Andrés Tirado-Sánchez

**Affiliations:** Dermatology Service, Mycology Department, Hospital General de México “Eduardo Liceaga”, Balmis 148, Colonia Doctores, CP: 03020. Cd. de México, México; atsdermahgm@gmail.com

**Keywords:** Sporotrichosis, disseminated cutaneous sporotrichosis, AIDS, *Sporothrix schenckii*, *Sporothrix brasiliensis*, amphotericin B, itraconazole

## Abstract

Sporotrichosis is an implantation or inoculation mycosis caused by species of *Sporothrix schenckii* complex; its main manifestations are limited to skin; however, cutaneous-disseminated, disseminated (visceral) and extracutaneous variants of sporotrichosis can be associated with immunosuppression, including HIV-AIDS, chronic alcoholism or more virulent strains. The most common extracutaneous form of sporotrichosis includes pulmonary, osteoarticular and meningeal. The laboratory diagnosis requires observing yeast forms and isolating the fungus; the two main causative agents are *Sporothrix schenckii* (*ss*) and *Sporothrix brasiliensis*. Antibody levels and species recognition by Polimerase Chain Reaction using biological samples or cultures are also useful. The treatment of choice for most cases is amphotericin B and subsequent itraconazole for maintenance therapy.

## 1. Introduction

Sporotrichosis is a subacute or chronic implantation (formerly subcutaneous) fungal infection, related to dimorphic fungi included within the *Sporothrix schenckii* complex [1,2,3,4]. The infection is commonly caused by inoculation of the fungus that lives in the soil, plants, and decaying material; therefore, disease is known as “rose gardener’s disease”; however, it may be transmitted by various animals (rodents); in Brazil, the infection is commonly transmitted by cats. Even though it is considered as a disease of global distribution, most reports are often observed in areas with tropical temperatures and climates [5,6,7] In Latin America: Brazil, Peru and Mexico reported the highest incidence [3,7,8,9,10].

Sporotrichosis is a polymorphic disease; its main clinical forms are cutaneous, the most frequent is cutaneous-lymphatic sporotrichosis, reported in up to 95% of cases; the second type is cutaneous-fixed sporotrichosis, reported in up to 30% [3,4,11], and occasionally it is the predominant form [12,13]; the third form is cutaneous-disseminated sporotrichosis, only reported in up to 8% of cases [3,4]. The extracutaneous forms are less commonly seen, and include disseminated sporotrichosis, pulmonary sporotrichosis and various osteoarticular, ocular and central nervous system disorders [4].

## 2. Brief Historical Background

Schenck reported the first case of sporotrichosis in 1898, while he was a medical student at Johns Hopkins Hospital (Baltimore) [14]; he described a classic case of cutaneous-lymphatic sporotrichosis and Smith isolated the fungus and identified it within the genus *Sporotrichum.* Later, Hektoen and Perkins [15] isolated the fungus from the exudates of skin lesions and classified it in the genus *Sporothrix.* de Beurmann, in France, described the first case and later, Gougerot reported more than 200 cases; they also described the first cases of cutaneous-disseminated sporotrichosis, in which they considered the fungus acted as an opportunist [4,16,17]. Marimon and Guarro et al., proposed that the etiology of sporotrichosis is a complex called *Sporothrix schenckii,* which includes five phylogenetically distinct species [18].

## 3. Etiology

For many years, *Sporothrix schenckii (sensu lato)* has been considered as the only etiologic agent of sporotrichosis; however, many strains display morphological variability (micro and macroscopically). Derived from studies of molecular biology, specifically based on gene sequences: chitin synthase, β-tubulin and calmodulin, species are placed into five clades: *Sporothrix brasiliensis* (Clade I); *Sporothrix schenckii* (Clade II); *Sporothrix globosa* (Clade III) *Sporothrix mexicana* (Clade IV) and *Sporothrix pallida* (formerly *S. albicans)* (Clade V) [4,18,19,20,21].

All species of the complex are dimorphic fungi; the first two exhibit the highest virulence rate. Other species result in sporadic cases, including *S. pallida*, which is considered a unique phytopathogen. Other species recently described and rarely associated with sporotrichosis in humans are *Sporothrix luriei* and *S. chilensis* [4,6,22,23,24]. A non teleomorph state has been reported, but an association with Ascomycetes of the genus *Ophiostoma* sp has been proposed; nevertheless, in a recent investigation, de Beer et al. separated the two genera, those that probably have a common origin; this has provided a solution to an old problem of fungal classification [25].

## 4. Epidemiology

### 4.1. Geographical Distribution

Sporotrichosis is the most frequent implantation mycosis; it has been reported worldwide, and although it is not a condition of compulsory reporting, it is a subject of extensive studies motivated by its incidence [4,7]. There are several geographic areas with the highest number of cases, for example, in Transvaal (South Africa), Simson (1947) reported one of the most important epidemics (3300 cases between 1941 and 1943); this was related to wood from mines, which was contaminated with *S. schenckii,* developing multiple pulmonary and cutaneous cases [16,26]. Other endemically important areas are in China, in the Northeast, Jillin Province, in the south in Guangdong [12]; in India in the north in the sub-Himalayan and Kangra regions and in Australia on the coast of New South Wales and the Southeast coast [7]. In Europe, there are few reports, mostly from people who traveled to endemic areas or by immigration; France, Italy and Spain report many of those cases [3,7].

In the United States, although this was where the first cases were reported, there are some series and isolated cases; the most important case series are related to the *moss sphagnum* (planting Bonsai) [3,4,27]; In general, the highest number of cases occurs in Latin America. A hyperendemic area has been reported in Peru (Andean region, Abancay) [9,10,28]; however, Brazil has the largest number of cases (Rio de Janeiro, Paraná, Rio Grande do Sul, Minas Gerais and São Paulo), because of the epidemic related to cats (zoonoses) [4,6,8,29]; in Mexico, high endemic areas can be found in Jalisco and Puebla [3,7]. Other countries with fewer but significant cases are: Colombia, Venezuela, Uruguay and Guatemala [7].

### 4.2. Habitat and Ecological Conditions

*Sporothrix schenckii* complex species usually live in warm, humid climates, with an average temperature of 20–25 °C and above 90% relative humidity; some may be thermo-resistant and others grow at pH ranging from 3.5 to 9.6 [3,4,12,20].

The largest number of infections occurs in the autumn and winter, where the relative humidity is higher in most endemic countries. *Sporothrix schenckii* (*sl*) lives in soil and environments high in cellulose, grasses, organic matter, wood, *sphagnum moss*, leaves and branches [3,4,19]. It has been isolated from different flowers and in countries like Mexico and China, has particular importance in relation to maize, i.e., the roots and leaves [4,12,30,31].

The disease can also be acquired from animals acting as indirect or passive vectors, since the fungus has been isolated from hooves and teeth. Rodents, like rats, mice and squirrels, are common vectors; cases of insects (ants, bees) and reptile’s bites, spiders and bats are also reported [3,4]. It is important to highlight the epidemic in Rio de Janeiro and other parts of southern Brazil, affecting domestic and stray cats (with a value of >4000), which has affected a significant part of the population (zoonoses) [6,8,29,31].

### 4.3. Entrance and Incubation Period

The main route of entry is cutaneous, through injury, wounds in contact with contaminated material and to a lesser extent by respiratory route, which thus provokes primary pulmonary cases [3,4]. It is believed that yeast propagation from cats is so intense that sometimes no trauma is detected. Cases of cutaneous-disseminated and disseminated sporotrichosis can be launched from a cutaneous or a pulmonary focus [4,32,33].

The incubation period depends on the size of the inoculums; for cutaneous cases, an average incubation time of three weeks is reported. In lung cases, it is uncertain, since most of cases are asymptomatic [3,33].

### 4.4. Occupation, Gender and Age

Sporotrichosis has been considered an occupational disease; it is mainly present in florists, hence the name of “Gardner’s disease” [5] or reed toxin, [12] peasants, housewives, school children (who do field work), hunters, miners, fishermen and especially in Brazil, veterinarians and cats and dogs caregivers are often at risk [2,3,4,5,6].

For all types of sporotrichosis, the gender ratio is 1:1, with a slight male predominance in particular taking into account most cutaneous-disseminated reports; however, in specific cases such as in the zoonotic epidemic in Rio de Janeiro, there is a 2:1 ratio of female: male, due to the predominance in housewives. Disseminated sporotrichosis (visceral) observed in male patients is more than 80%; a possible explanation relies in the fact that the majority of HIV-AIDS-related cases are seen in male patients [3,4,34,35,36].

Most reports point out that two-thirds of the cases with sporotrichosis are young adults, mostly between 16 and 35 years old and only one-third occurs in children (5–15 years) [2,3,37]. For cases of cutaneous-disseminated and disseminated sporotrichosis, most cases occur in adults and are exceptional in children [4,37].

## 5. Predisposing Factors

Particularly, for cutaneous-disseminated and disseminated sporotrichosis, more cases are diagnosed in immunocompromised patients, mostly related to HIV-AIDS [3,34,35,36], chronic alcoholism [28,38,39,40,41], hematologic cancer (leukemia and lymphomas) [42,43,44], Diabetes mellitus [3,4,45], steroid treatment [2,3,46], transplanted patients [47,48] pregnancy [3,49] (although may be considered immunocompetent), malnutrition [3,4] and exceptional cases are reported for immunocompetent patients [49,50,51,52]. In the particular case of pregnant patients, some authors do not consider this a predisposing factor to develop disseminated sporotrichosis; however, in our experience, the majority of cases presented as disseminated cutaneous sporotrichosis [3].

## 6. Pathogenesis

Primary cutaneous sporotrichosis is often initiated through trauma with contaminated material; the primary lesion occurs at the site of inoculation, in the form of a chancre (an ulcerated nodule). Two or three weeks later, an immune response involving CD4+ T-lymphocytes, macrophages, dendritic cells and neutrophils is essential for infection control and/or inoculum stabilization; this cell infiltrate later develops a granulomatous reaction [53]. INF-γ activates the Th1-type response, enhancing macrophage functions [54,55].

We believe that an immunosuppressant status is essential for cutaneous-disseminated and disseminated sporotrichosis. However, Zhang et al. [56] compared cutaneous-disseminated versus cutaneous-lymphatic and cutaneous-fixed sporotrichosis cases and showed a variation in the 10-bp deletion genotype in the ribosomal NTS region, suggesting a more virulent strain that may produce cutaneous-disseminated and disseminated sporotrichosis [4,31,57].

Pulmonary sporotrichosis is launched by primo-contact with the fungus, which needs an important inoculum; this results in an asymptomatic, limited pneumonic disease, and later, depending on the immune status, may be the focus of systemic spread [4,33].

There have been small differences in pathogenicity within *S. schenckii* complex species, for example, *S. brasiliensis* is more virulent than *S. schenckii (ss)*, but both produce almost the same clinical picture. The main virulence factors are: fungal dimorphism, thermotolerance, melanin production (conidia), extracellular proteins (enzymes such as glycoproteins, phosphatases), epithelial adhesion and the antigenic presence of the l-rhamnose substance (part of the peptide ramnomanan) [3,4,31,56,57].

## 7. Clinical Features

### 7.1. Cutaneous Sporotrichosis

Cutaneous sporotrichosis comprises three distinct clinical forms: cutaneous-lymphatic sporotrichosis is the classic and most common presentation; it is usually located in upper limbs, lower limbs and the face; it is formed by linearly distributed, painful or pruriginous ulcerated nodules that in chronic stages may develop verrucous plaques [1,2,3,4,11]. The second form is cutaneous-fixed sporotrichosis, which occurs in the same inoculation site (called sporotrichoid chancre), usually consisting of an asymptomatic, sole, vegetative, or slow-growing verrucous lesion, and a squamous, erythematous or violaceous halo [1,2,3,4,12,13].

**Figure 1:**
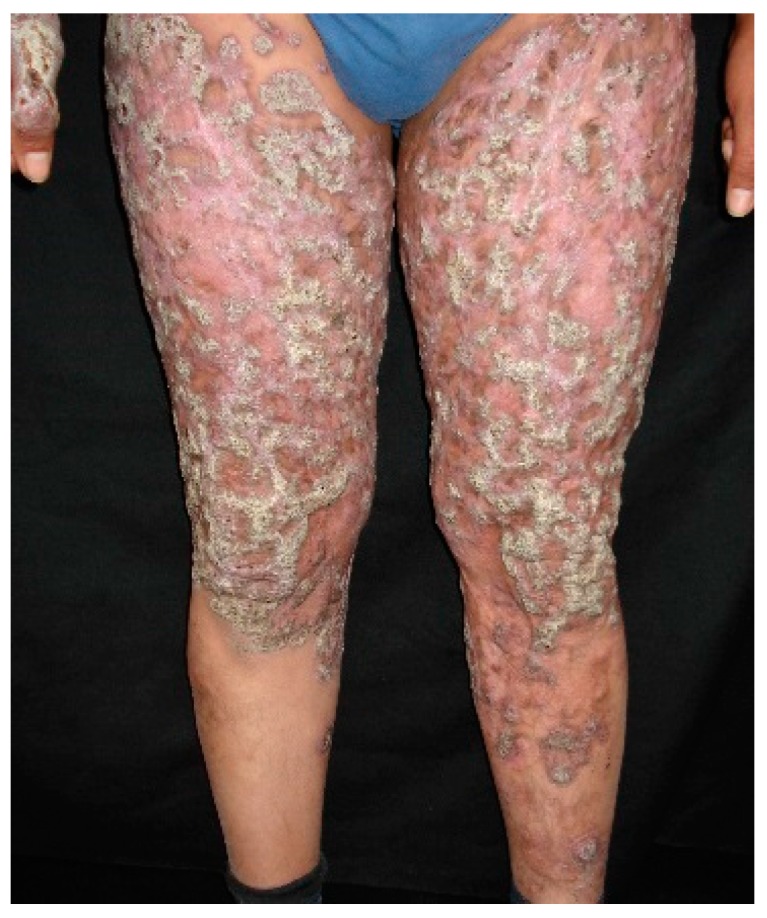
Extensive cutaneous disseminated sporotrichosis associated to chronic alcoholism.

Cutaneous-disseminated sporotrichosis, also called hematogenous sporotrichosis, is a rare entity, usually seen in immunocompromised patients [3,4,46], due to the mentioned predisposing factors, where the causative agent has a role as an opportunist; there are few reports in immunocompetent patients [49,50,51,52], pointing out that virulence is an important factor for disease development; however, this theory has not been fully verified [56,57]. Clinical manifestations of cutaneous-disseminated sporotrichosis include ulcerated nodules and verrucous plaques (Figure 1) [58,59,60]; there are cases of many inoculations, this may be related to cat scratches (most cases reported in Brazil) and may develop in immunocompetent patients [2,4,29]. Cutaneous-disseminated sporotrichosis can be found affecting any part of the body surface, and even mucous membranes (mouth, pharynx, penis glans) in one third of the patients, developing ulcerations and sinus plaques [34,35,36,61]. It may affect bones and joints, producing small granulomatous lesions or even extensive lytic lesions and osteomyelitis, associated with joint effusions, edema and severe pain; the most affected bones include tibia, carp and metacarpus, ulna, knee and ankle, in that order [4,41,62,63,64,65]. Osteoarticular cases or sporotrichoid arthritis without cutaneous involvement, derived from pulmonary or hematogenous dissemination, has been reported [4,33]. Cutaneous-disseminated sporotrichosis can extend to various organs and systems (e.g., testes, central nervous system, etc.) rapidly progressing to fungemia [3,4,34,35,38].

### 7.2. Extracutaneous Sporotrichosis

Pulmonary sporotrichosis is a rare entity; about 100 cases have been reported so far [33,66,67], most of them are primary disease and they are usually seen in high endemic areas. It is classified into two clinical types 1. The chronic type (most common), usually asymptomatic (98%), it presents with limited cavitary zones, indistinguishable from tuberculosis; symptomatic cases manifest as pneumonia, with little cough and expectoration. The radiographs show areas of condensation, or infiltrated milliar type 2. The acute and progressive type, involves tracheobronchial lymph nodes, developing massive adenopathies, which may derive into bronchial obstruction; common symptoms include cough with abundant expectoration, dyspnea and fatigue. Chest X ray shows parahilar lymphadenopathy and, less commonly, mediastinal enlargement. Aung et al., [32] reported 86 cases diagnosed during 50 years (1960–2010), 74.4% of those were primary, while 25.6% were multifocal and most cases involved immunocompromised patients.

Central nervous system involvement is one of the deadliest complications of sporotrichosis. It has been reported in patients with severe immunosuppression often related to leukemia and post-transplanted therapy; however, the largest numbers of cases are associated with HIV-AIDS [34,36,68] and as part of zoonotic epidemics. These cases are commonly due to *S. brasiliensis*; invasion seems to occur in about 17% of cases; however, the exact frequency is unknown [36]. Central nervous system involvement manifests as meningoencephalitis and hydrocephalus, clinical symptoms include headache, fever, neck stiffness, mental confusion and vomiting; the main differential diagnosis is cryptococcosis [4,67]. It is also important to mention that because of the antiretroviral therapy, immune reconstitution inflammatory syndrome can be present; it is estimated that it occurs in little more than 7% and there can be diverse clinical manifestations [34,36,68,69].

Disseminated sporotrichosis can affect the skin, lungs, sinuses (sinusitis), liver, kidney, eyes (uveitis, endophthalmitis), genitalia, heart (endocarditis). Clinical features are variable and are often detected at necropsy [3,4,32,36,39,41,48].

## 8. Laboratory Diagnosis

**Figure 2:**
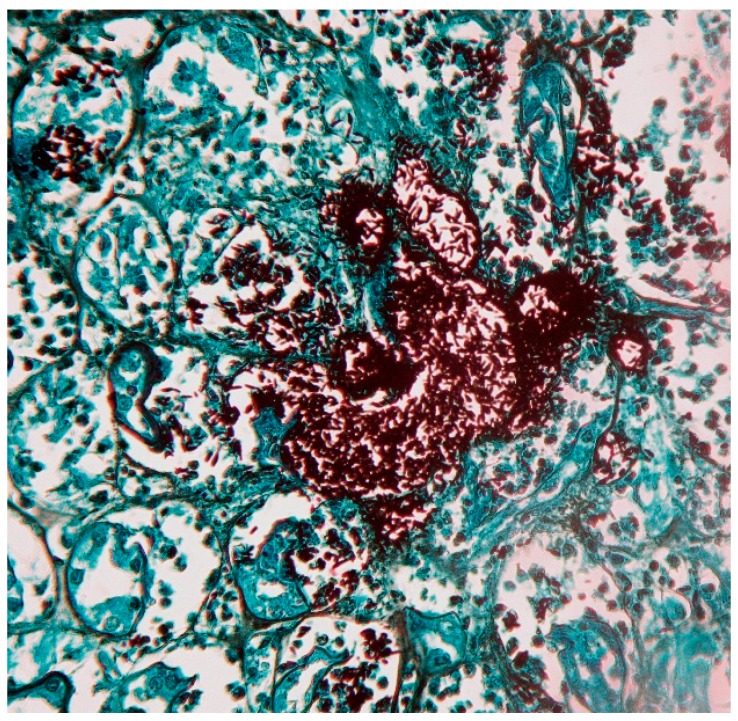
Biopsy of disseminated sporotrichosis. Renal biopsy with multiple clusters of lengthened yeast forms “cigar-shaped” (Grocott, 40×).

Direct examinations and staining are not useful for diagnosis of cutaneous-lymphatic and cutaneous-fixed sporotrichosis, since yeasts are observed only in a low percentage (5%–10%), whereas in cases of cutaneous-disseminated, disseminated and pulmonary sporotrichosis, Gram, Giemsa, Periodic Acid–Schiff (PAS) and Gomori-Grocott stains are useful for diagnosis as well as immunofluorescence techniques [3,4,11,70]. Yeast forms are usually round, oval or lengthened, described as “cigar-shaped” (Figure 2); in cases of immunocompromised patients, large clusters of yeast are observed, similar to feline sporotrichosis [8,29]. Differential diagnosis includes mainly histoplasmosis; in fungemia [3,4,35,36], yeasts are easily observed from blood imprints and no special staining is required [4,38,71,72].

**Figure 3:**
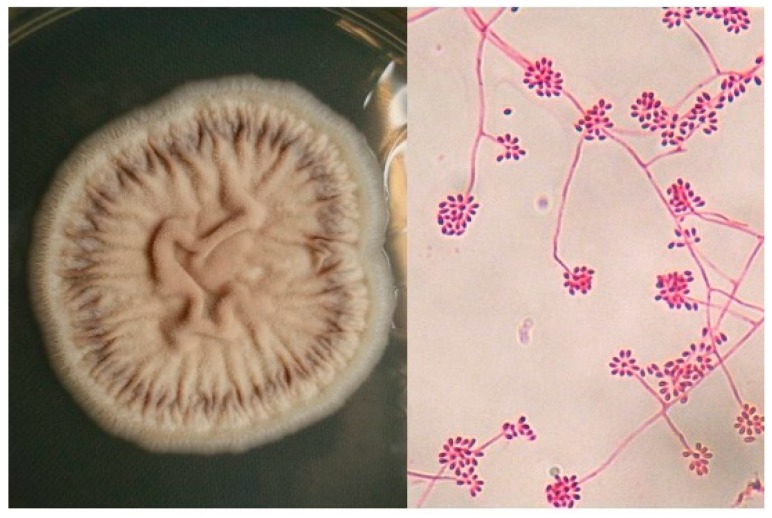
Culture of *Sporothrix schenckii* (Sabouraud media, 28 °C) Filamentous state with thin hyphae and denticle microconidia like “daisy flowers” (Erythrosine, 40×).

Cultures from exudative lesions, scale, tissue fragment, sputum and blood are the gold standard for diagnosis. Sabouraud dextrose agar with and without antibiotics, incubated at 28 °C is often useful; the colonies may develop between 5 and 8 days; because of its dimorphic feature, *Sporothrix* spp. can produce yeast colonies (blood agar, chocolate agar, BHI agar) in rich media, incubated at 37 °C; this must be distinguished from bacterial colonies. *S. schenckii* (*sl*), presents filamentous colonies with thin 1–3 micron septate, branched, hyaline hyphae, reproduce by ovoid, round and pyriform microconidia, derived from the denticle (sympudolic) form of conidiophores (10 to 30 μm in length) or directly from the hyphae; microscopically appear as “peach blossoms or daisies” (Figure 3) [3,4,11,24].

The histopathology offers a useful tool for cutaneous-lymphatic and cutaneous-fixed sporotrichosis diagnosis. Suppurative granulomatous pictures (84%) [70], where dispersed yeasts can rarely be seen and often with a radiated halo (asteroid bodies) are usually reported. In cases of cutaneous-disseminated and disseminated sporotrichosis, a similar histological distribution is seen as well as the presence of yeasts, depending on the patient’s immune status; some cumulus to large amounts of yeasts can also be noted, most of them are round or elongated; this becomes obvious with PAS and Grocott stains. It must be distinguished from histoplasmosis [3,70].

Other useful tests are precipitins, agglutinins and complement fixation [3,4,73]; the intradermal reaction with sporotrichin may be useful in cutaneous-lymphatic and cutaneous-fixed sporotrichosis, but not in cases where it is usually negative. To identify the fungus in tissues, exudates and culture, PCR and PCR-RFLP (chitin-synthetase gene, *ChS1*, 26S rDNA gene and topoisomerase II gene) are helpful. ELISA with the antigen SsCBF is usually a good diagnosis and prognosis method, especially when no clinical lesions can be assessed [4,18,20,74,75,76,77].

## 9. Treatment

Various species in the *S. schenckii* complex have different in vitro susceptibility to various systemic antifungals [3,4,77].

For cutaneous-lymphatic and cutaneous-fixed sporotrichosis, potassium iodide can be administered in diluted solutions or in drops of saturated solution with favorable response. According to the treatment guidelines, the drug of choice for both clinical forms is itraconazole at 200 mg/day for 3–6 months [2,3,4,11,78,79].

For cutaneous-disseminated, disseminated, pulmonary and osteoarticular sporotrichosis, according to the treatment guidelines [80], the drug of choice is amphotericin B, preferably lipidic at 3–5 mg/kg/day and if the deoxycholate form is used, recommended doses are 0.7 to 1 mg/kg/day; the treatment duration varies depending on the response and side effects (mainly renal damage) [79,80]. After intensive and aggressive treatment, the most useful maintenance therapy is itraconazole at 400 mg/day (divided in two doses) with variable response. In cases associated with HIV-AIDS or immunosuppressed patients, it can be administered at 200 mg/day for a long time to avoid relapses [3,34,36]. For fungemia and central nervous system involvement, high doses of amphotericin B (lipid 5 mg/kg/day and deoxycholate 1 mg/kg/day) are usually needed, continuing with itraconazole between 200 and 400 mg/day for at least one year [4,80,81].

In HIV/AIDS-associated sporotrichosis, therapy usually varies when compared with immunocompetent patients. Freitas et al., [34] reported 21 patients with HIV-AIDS; 12/21 (57%) had cutaneous-disseminated or disseminated sporotrichosis; 10/21 were treated with amphotericin B alone or with itraconazole and 11 patients used itraconazole as monotherapy at 100–400 mg/day. Two patients died (9.52%) and the rest (90.48%) reached clinical and mycological cure; in another review, the death rate was estimated as high as 30.2% [36]. 

We consider that, if cutaneous-lymphatic or cutaneous-fixed sporotrichosis develops in HIV-AIDS patients, itraconazole should be started as monotherapy; however, for the disseminated forms, it is necessary to initiate amphotericin B and eventually continue with itraconazole. In the later cases, the response to treatment depends not only on antifungal therapy but also on antiretroviral therapy (HAART), and the risk of drug interactions such as itraconazole with ritonavir and indinavir, or amphotericin B with tenofovir should be highlighted [34,36]. Terbinafine can also be used with less efficacy when compared with itraconazole, but with fewer adverse effects and drug interactions than triazoles [3,80,81,82,83].

The recommended amphotericin B dose in children is 0.7 mg/kg/day and itraconazole 6–10 mg/kg/day [37,80]. Among cutaneous-osteoarticular cases, we have observed a successful response when combining itraconazole and/or potassium iodide plus sulfamethoxazole/trimethoprim (800 mg/160 mg) twice daily [3].

Importantly, *S. schenckii (sl)* is temperature-sensitive and temperatures above 42 °C often inhibit its growth; thermotherapy or local heat (hyperthermia) with temperatures of 45 °C has been used, as well as hot baths at 45 °C for 15 to 20 min, 2 to 3 times a day with significant improvement. This therapy is not recommended as monotherapy, but an adjuvant therapy and only for localized cases or in cases of cutaneous-lymphatic sporotrichosis [3,4,37,80].

In cases of sporotrichosis in pregnant patients, many drugs cannot be administered because of their potential teratogenicity, and thus represent a challenge for the clinician [4,49,80]. During the third trimester of gestation with few cutaneous lesions, thermotherapy is an acceptable alternative. During postpartum and a short lactation period, treatment with amphotericin B or itraconazole can be recommended; however, in severe cases with large dissemination, the drug of choice is lipidic amphotericin B (3–5 mg/kg/day) or the deoxycholate form (0.7–1 mg/kg/day), which can be complemented with local hyperthermia. Orfino-Costa et al. [84] reported five cases of sporotrichosis during pregnancy; four corresponded to cutaneous-lymphatic sporotrichosis and 1 to cutaneous-fixed sporotrichosis; amphotericin B was used in 2 cases, achieving mother’s cure and a healthy newborn; in two more patients, local heat was used, the mothers cured but one of the newborn died shortly after birth. A patient used terbinafine without prescription, with good clinical response for both mother and child; however, this drug has a B-category warning note for pregnancy.

There are few reports about amphotericin B resistance, which can be solved by single administration of itraconazole at high doses [81]; there are also reports associating amphotericin B with posaconazole with good results [44]. With the new triazoles, there are no case series only some reports, for example, posaconazole has been combined with other antimycotics and there is good response in vivo (mice); whereas voriconazole has little response in vitro and in vivo and there are no reports in humans [85,86,87,88].

## 10. Conclusions

Cutaneous-disseminated, disseminated and pulmonary sporotrichosis are rare entities occurring in less than 10% of cases; however, in patients with severe immunodeficiency (HIV/AIDS), sporotrichosis can lead to poor prognosis. Unlike cutaneous-lymphatic and cutaneous-fixed sporotrichosis, it is possible to observe yeasts and isolate the fungus; the most common isolated agents are *S. schenckii* (*ss*) and *S. brasiliensis*. The treatment of choice is amphotericin B, followed by itraconazole and in cases of severe immunosuppression, long-term maintenance treatment with itraconazole is usually required (Table 1).

## Figures and Tables

**Table 1:** Main differences between the types of sporotrichosis.

Variable	Cutaneous Lymphatic and Cutaneous Fixed Types [1,2,3,4,5,9,10,11,12,13,28,37,79,80]	Cutaneous-Disseminated, Disseminated and Pulmonary Types [1,2,3,4,5,12,13,30,31,32,33,34,35,36,37,38,39,40,42,43,44,45,46,47,48,49,50,51,52,58,59,60,61,62,63,64,65,66,67,68,69,70,71,72,73,74,75,76,77,79,80,81,82,83,84]
Main etiological agents	*S. schenckii (sl) & S. brasiliensis*	*S. schenckii (sl) & S. brasiliensis*
Gender proportion Male:Female	1:1, with slight male predominance.	8:2 Male predominance especially by association with HIV/AIDS.
Age group	Mainly in young adults (2/3) and children (1/3)	Mostly in adults and rare in children.
Predisposing factors	Primarily immunocompetent patient.	HIV/AIDS, chronic alcoholism, diabetes, hematologic cancer, steroid treatment, pregnancy and rare in immunocompetent patients.
Location	Mainly in upper limbs; in children on the face and limbs	The cutaneous form is present throughout the body. Extracutaneous manifestations are common (lungs, meningeal and osteoarticular)
Laboratory diagnosis	Yeast forms are not commonly seen (only 5%–10%). Asteroid bodies are seen. Gold standard: culture. Positive sporotrichin (100% of cases)	Yeast forms are easily seen (100%). Clusters of round and lengthened yeast forms are noted. Gold standard: culture. Sporotrichin is usually negative.
Treatment/time	Itraconazole Potassium iodide From 3 to 6 months.	Initial: Amphotericin B Intensive: Amphotericin B + Itraconazole. Maintenance: Itraconazole 6–12 months.
Outcome	Good	Bad. Average death (HIV/AIDS) 30%.
